# Single-shot incoherent digital holography based on generalised three-step phase-shifting method with 1D phase grating

**DOI:** 10.1038/s41598-025-90793-8

**Published:** 2025-03-08

**Authors:** Teruyoshi Nobukawa, Masahiro Usui, Masahide Goto, Nobuhiro Kinoshita, Kei Hagiwara, Tetsuhiko Muroi

**Affiliations:** Japan Broadcasting Corporation (NHK), Science and Technology Research Laboratories, Kinuta 1-10-11, Setagaya, Tokyo, 157-8510 Japan

**Keywords:** Applied optics, Imaging and sensing, Optical metrology

## Abstract

Incoherent digital holography (IDH) is a passive holographic 3D imaging technique under spatially incoherent light. Single-shot recording with high light-utilization efficiency and high spatial resolution is a challenge in IDH. Herein, we propose a single-shot three-step phase-shifting IDH system with a 1D phase grating that has a line-and-space structure. The 0th and ± 1st diffraction orders of the grating are used for simultaneously creating three self-interference holograms, which are then captured using an image sensor at a single exposure. Three-step phase shifts are introduced into the holograms using the geometric phase of circular polarizations. To compensate the intensity variations between the holograms due to grating fabrication errors, we derive and implement a generalised three-step phase-shifting algorithm. We experimentally performed the single-shot recording of reflective objects as a proof-of-principle demonstration. We further examined the theoretical noise tolerances between three- and four-step phase-shifting methods for grating-assisted space-division phase shifting. The results show that the three-step phase-shifting method is superior to the four-step method when the diffraction efficiency of the 1D phase grating is at least four-thirds higher than that of the 2D phase grating used for the four-step method.

**Supplementary Information:**

The online version contains supplementary material available at 10.1038/s41598-025-90793-8.

## Introduction

In incoherent digital holography (IDH), holograms are recorded digitally under spatially incoherent light through self-interference and reconstructed via diffraction calculation^1–3^. Unlike conventional digital holography, which involves the use of laser^4^, IDH facilitates passive 3D imaging under ambient lights, such as sunlight, light-emitting diode (LED) lighting, and fluorescent lights^1–3^, via their self-interference. This IDH functionality has expanded the application of digital holography to methods such as outdoor 3D imaging^4^, fluorescence microscopy^5^, and thermal radiation measurement^6^.

Similar to conventional digital holography, which requires laser^7^, IDH suffers from image degradation caused by bias and conjugate terms in a recorded hologram. These are digitally removed by conventional IDH setups using time-division phase-shifting methods^4,8^. A series of holograms with different phase shifts is captured sequentially and processed using a phase-shifting algorithm^9^. Although the time-division phase-shifting method can simply yet powerfully obtain high-quality reconstructed images^1–5^, its need for sequential recording leads to difficulties in recording dynamic phenomena and moving objects in IDH. Some researchers addressed this problem by introducing temporal phase shifts at high speeds using stacked ferroelectric liquid crystal devices^10^. Compressive-sensing-based reconstruction is also an effective approach to recording dynamic phenomena and objects^11,12^. This approach infers object information from limited measurements by relying on sparseness in some information domains, reducing the number of holograms needed to retrieve complex amplitude distributions. However, the detectable temporal and/or space bandwidth products of the objects to be captured of the abovementioned approaches^10–12^ are limited.

Single-shot phase shifting is a promising method for uniquely retrieving complex amplitude distributions without restrictions on object information. Several single-shot phase-shifting methods and geometries have been proposed and developed, such as pixelated-mask-based and grating-based methods, as briefly reviewed below. In a pixelated-mask-based method, a polarization-sensitive image sensor^13^is often used to capture holograms with different phase shifts simultaneously. This image sensor incorporates a linear-polarizer array whose single fundamental unit consists of 2 × 2 pixels with 0-, 45-, 90-, or 135-degree-rotated linear polarizers. When a pair of orthogonal circular polarizations is incident on the linear-polarizer array, four holograms with different phase shifts can be created, namely, 0, π/2, π, and 3π/2, according to the concept of a geometric phase. Four individual holograms can be acquired by capturing and demosaicing a single image. Polarization-sensitive image sensors can effectively implement single-shot recording in IDH^14–22^. A wave-plate array can also be used in place of the linear-polarizer array for single-shot recording^23^. However, the selection of sensor specifications for pixelated-mask-based phase shifting is currently limited. Because the sensor specifications, such as pixel pitch, number of pixels, quantization level, and noise tolerance, affect the spatial resolution, quality, and field of view in IDH^24^, they should be selected according to application. Moreover, a pixelated-mask-based method halves the spatial resolution of a detectable complex amplitude distribution via the demosaicing process. By contrast, a grating-based method eliminates the need for demosaicing. This approach uses diffraction from a grating to create holograms^25^. Binary checkerboard gratings (hereinafter called 2D phase gratings) have been adopted in IDH geometries^25–27^. A 2D phase grating has horizontal and vertical periodicities, so its Fourier spectrum consists of four main peaks. With this 2D phase grating, four holograms can be created individually at different segmented areas of an image sensor. Thus, a grating-based method eliminates the need for a polarization-sensitive image sensor and enables flexible adaption of any image sensor in principle. An image sensor must be selected such that imaging performance is optimized, depending on the application. However, previous grating-based methods have low light-utilization efficiency. The diffraction efficiency of each of the four main peaks is 16.4% in theory^25–27^. Thus, only 65.6% (= 16.4 × 4) of the input light energy contributes to the creation of holograms. The remaining light energy is wasted as high diffraction orders. This low light-utilization efficiency reduces the signal-to-noise ratio (SNR) during hologram detection using an image sensor.

Herein, we propose a single-shot geometry based on a generalised three-step phase-shifting method with a 1D phase grating to achieve higher light-utilization efficiency compared with that of the conventional geometry (with a 2D phase grating)^25–27^. The 1D phase grating has a line-and-space structure, thereby enabling binary phase modulation. We use the 0th and ± 1st diffraction orders of the 1D phase grating to create three holograms and implement a three-step phase-shifting algorithm. Because the grating has 1D periodicity, its diffraction peaks appear along a single direction in the Fourier space. Thus, the 1D phase grating may achieve higher light-utilization efficiency than the 2D phase grating^25–27^. In a study on coherent interferometry, a similar 1D phase grating was used for single-shot three-step phase shifting with a Michelson interferometer^28^. In this method, three-step phase shifts were induced based on a dynamic phase. By contrast, we use a geometric phase instead of a dynamic phase by interfering orthogonal circular polarizations through a linear polarizer. Moreover, we design an optical geometry for creating self-interference holograms using a common-path interferometer.

The rest of this article is organized as follows. First, we describe the principle of single-shot IDH based on generalised three-step phase shifting with a 1D phase grating. Next, we show the design of the 1D phase grating for three-step phase shifting and then evaluate the diffraction efficiency of a fabricated 1D phase grating. Afterward, we experimentally demonstrate single-shot recording through the proposed method. Finally, we theoretically compare the noise tolerance of three- and four-step phase-shifting methods for grating-assisted space-division phase shifting.

## Principle

**Fig. 1:**
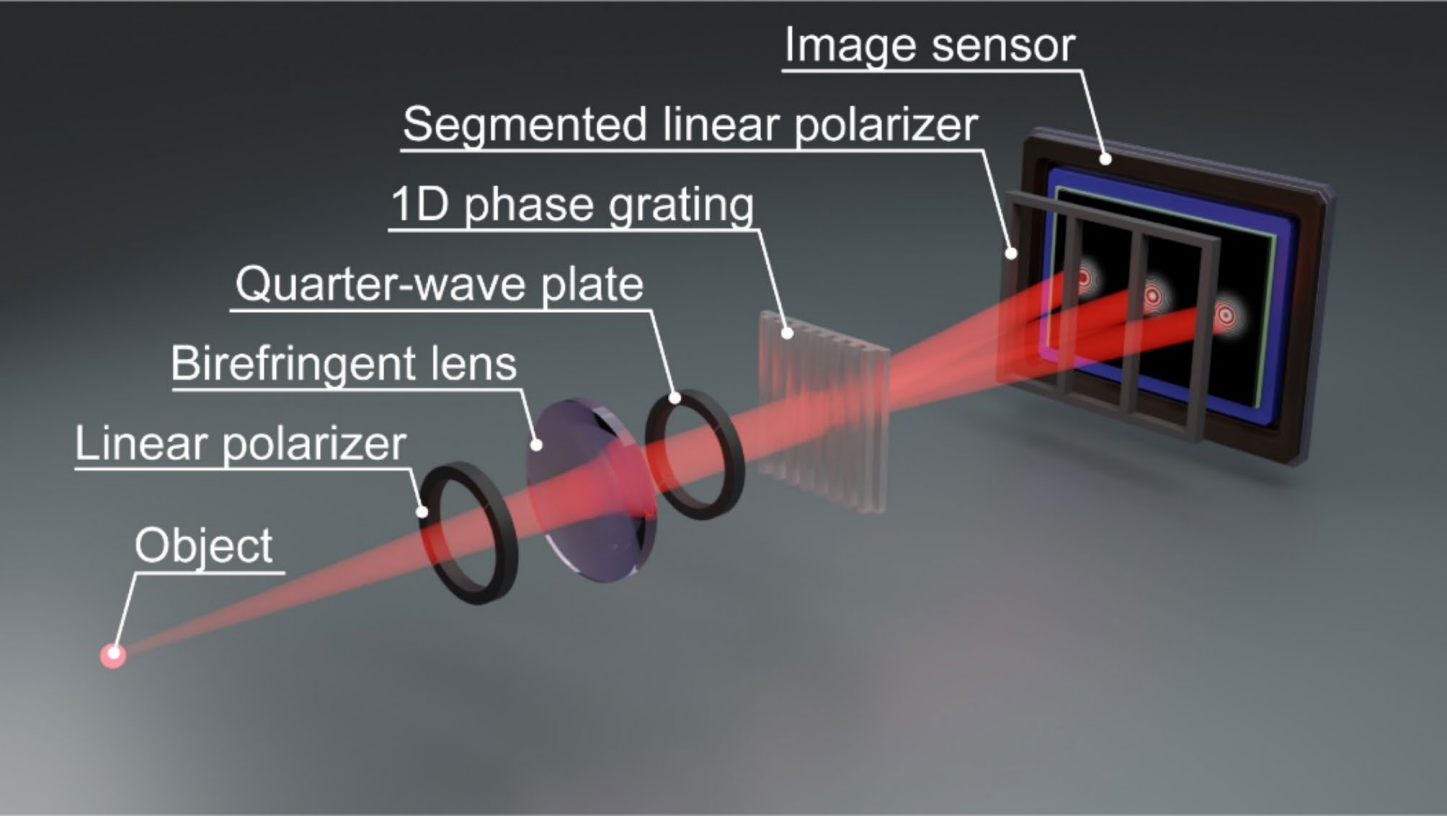
Schematic of single-shot geometry based on generalised three-step phase-shifting method with 1D phase grating.

Figure 1 is a schematic of the proposed method. This geometry is based on a common-path interferometer with orthogonal polarizations. An object with spatially incoherent light can generally be regarded as a collection of infinitesimal point sources. For brevity, we describe the recording process of the self-interference hologram of a single point source centred at the optical axis with a quasimonochromatic wavelength $\lambda$. The propagated light from the point source is first polarized 45 degrees with respect to the fast axis of the subsequent birefringent lens. The birefringent lens has dual focal lengths ${f}_{1}$ and ${f}_{2}$for each orthogonal linear polarization. According to Fresnel diffraction theory^29^, each complex amplitude distribution ${u}_{1}(x,y)$ or ${u}_{2}(x,y)$ of orthogonal linear polarization immediately across the plane immediately behind the birefringent lens is

1 $$\begin{array}{c}{u}_{1,2}=C\text{exp}\left\{\frac{i\pi}{\lambda}\left(\frac{{f}_{\text{1,2}}-{z}_{s}}{{z}_{s}{f}_{\text{1,2}}}\right)\left({x}^{2}+{y}^{2}\right)\right\}\end{array}$$

where ${z}_{s}$ is the distance between the object and the birefringent lens and $C$ is a complex constant. To make use of the geometric phase of the circular polarizations, we use the quarter-wave plate to convert the pair of orthogonal linear polarizations into a pair of orthogonal circular polarizations. The co-propagated lights are incident on the 1D phase grating. Because of its line-and-space structure, the grating introduces binary phase distribution on the incident lights. The design and characteristics of the grating are described in the next section. We leverage the 0th and ± 1st diffraction orders of the 1D phase grating to create three self-interference holograms on an image sensor plane positioned at a distance ${z}_{h}$ from the birefringent lens. The diffracted optical field ${u}_{d1}(x,y)$ or ${u}_{d2}(x,y)$ of each circular polarization on the image sensor plane is

2 $${u}_{d1,d2}=\left\{\sqrt{{\eta}_{-1}}\delta\left({z}_{h}\text{tan}\alpha,0\right)+\sqrt{{\eta}_{0}}\delta\left(\text{0,0}\right)+\sqrt{{\eta}_{+1}}\delta\left({-z}_{h}\text{tan}\alpha,0\right)\right\}\begin{array}{c}*{C}_{\text{1,2}}^{{\prime}}\text{exp}\left\{\frac{i\pi}{\lambda}\left(\frac{{f}_{\text{1,2}}-{z}_{s}}{{z}_{s}{f}_{\text{1,2}}+{z}_{h}{f}_{\text{1,2}}-{z}_{s}{z}_{h}}\right)\left({x}^{2}+{y}^{2}\right)\right\},\end{array}$$

where ${\eta}_{-1}$, ${\eta}_{0}$, and ${\eta}_{+1}$ are the diffraction efficiencies of the 0th and ± 1st diffraction orders for the 1D phase grating; $\delta\left(\dots\right)$ is the Dirac delta function; $\alpha$ is the diffraction angle of the 1D phase grating, which is determined by the grating period; and $*$is the 2D convolution operator. This equation is effective under the assumption that the 1D phase grating functions on the basis of scalar diffraction theory^29^. The polarization states of ${u}_{d1}$ and ${u}_{d2}$ are orthogonal to each other, so they cannot create holograms. Moreover, no phase shift occurs between the diffracted lights from the 1D phase grating. To create holograms with different phase shifts, we introduce a segmented linear polarizer between the 1D phase grating and the image sensor. The segmented linear polarizer consists of three linear polarizers whose transmission angles are rotated at $\theta=$ 0, 60, and 120 degrees. The polarization states of the diffracted lights ${u}_{d1}$ and ${u}_{d2}$ are therefore aligned with the common linear polarization after the lights pass though the segmented linear polarizer, generating holograms. The phase-shift amount $\varphi$ on the holograms is determined by the transmission angle of the linear polarizer according to the geometric phase of the circular polarization, or $\varphi=2\theta$. Consequently, the image sensor simultaneously captures the following holograms as a single image:

3 $$\begin{array}{c}{I}_{-1}={\eta}_{-1}{\left|{C}_{1}^{{\prime}}\text{exp}\left\{\frac{i\pi}{\lambda}\left(\frac{{f}_{1}-{z}_{s}}{{z}_{s}{f}_{1}+{z}_{h}{f}_{1}-{z}_{s}{z}_{h}}\right)\left({x}^{2}+{y}^{2}\right)\right\}+{C}_{2}^{{\prime}}\text{exp}\left\{\frac{i\pi}{\lambda}\left(\frac{{f}_{2}-{z}_{s}}{{z}_{s}{f}_{2}+{z}_{h}{f}_{2}-{z}_{s}{z}_{h}}\right)\left({x}^{2}+{y}^{2}\right)+{\varphi}_{-1}\right\}\right|}^{2},\end{array}$$

4 $$\begin{array}{c}{I}_{0}={\eta}_{0}{\left|{C}_{1}^{{\prime}}\text{exp}\left\{\frac{i\pi}{\lambda}\left(\frac{{f}_{1}-{z}_{s}}{{z}_{s}{f}_{1}+{z}_{h}{f}_{1}-{z}_{s}{z}_{h}}\right)\left({x}^{2}+{y}^{2}\right)\right\}+{C}_{2}^{{\prime}}\text{exp}\left\{\frac{i\pi}{\lambda}\left(\frac{{f}_{2}-{z}_{s}}{{z}_{s}{f}_{2}+{z}_{h}{f}_{2}-{z}_{s}{z}_{h}}\right)\left({x}^{2}+{y}^{2}\right)+{\varphi}_{0}\right\}\right|}^{2},\end{array}$$

5 $$\begin{array}{c}{I}_{+1}={\eta}_{+1}{\left|{C}_{1}^{{\prime}}\text{e}\text{xp}\left\{\frac{i\pi}{\lambda}\left(\frac{{f}_{1}-{z}_{s}}{{z}_{s}{f}_{1}+{z}_{h}{f}_{1}-{z}_{s}{z}_{h}}\right)\left({x}^{2}+{y}^{2}\right)\right\}+{C}_{2}^{{\prime}}\text{exp}\left\{\frac{i\pi}{\lambda}\left(\frac{{f}_{2}-{z}_{s}}{{z}_{s}{f}_{2}+{z}_{h}{f}_{2}-{z}_{s}{z}_{h}}\right)\left({x}^{2}+{y}^{2}\right)+{\varphi}_{+1}\right\}\right|}^{2}.\end{array}$$

By cropping the three holograms from the single captured image, we can implement a phase-shifting algorithm to retrieve complex amplitude distributions. With an ideal 1D phase grating (designed and fabricated such that ${\eta}_{-1}={\eta}_{0}={\eta}_{+1}$), a traditional three-step phase-shifting algorithm can be adopted^30,31^. However, actual 1D phase gratings have fabrication errors. This leads to variations in the diffraction efficiencies between the 0th and ± 1st diffraction orders, and the three hologram intensities differ from each other. In addition, the phase-shift amounts on holograms may be affected by the misalignment of optics. If we retrieve a complex amplitude distribution from unbalanced holograms using a traditional three-step phase-shifting algorithm, sufficiently reducing the undesired twin-image and bias terms of the holograms will be difficult. Given an imperfect grating, we robustly retrieve a complex amplitude distribution $o$ by deriving a generalised three-step phase-shifting algorithm.

6 $$\begin{array}{c}o=\frac{\left[1-\text{exp}\left\{i\left({\varphi}_{-1}-{\varphi}_{0}\right)\right\}\right]\left({\eta}_{0}{\eta}_{-1}{I}_{+1}-{{\eta}_{+1}{\eta}_{-1}I}_{0}\right)+\left[1-\text{exp}\left\{i\left({\varphi}_{+1}-{\varphi}_{0}\right)\right\}\right]\left({\eta}_{+1}{\eta}_{-1}{I}_{0}-{\eta}_{0}{\eta}_{+1}{I}_{-1}\right)}{-2i{\eta}_{+1}{\eta}_{-1}\left\{\text{sin}\left({\varphi}_{+1}-{\varphi}_{0}\right)+\text{sin}\left({\varphi}_{-1}-{\varphi}_{+1}\right)+\text{sin}\left({\varphi}_{0}-{\varphi}_{-1}\right)\right\}}.\end{array}$$

When ${\eta}_{-1}={\eta}_{0}{=\eta}_{+1}$, ${\varphi}_{0}=0$, ${\varphi}_{+1}=2\pi/3$, and ${\varphi}_{-1}=4\pi/3$, the above equation turns into the traditional three-step phase-shifting algorithm. From the retrieved complex amplitude, we can obtain a reconstructed image ${o}_{r}$ of the recorded object at an arbitrary reconstruction distance ${z}_{r}$using a numerical back-propagation method, such as an angular spectrum method^29^.

7 $$\begin{array}{c}{o}_{r}\left(x,y\right)={FT}^{-1}\left[FT\left[o\left(x,y\right)\right]\text{e}\text{x}\text{p}\left\{i2\pi{z}_{r}\sqrt{{\lambda}^{-2}-{\mu}^{2}-{\nu}^{2}}\right\}\right],\end{array}$$

where $FT\left[\dots\right]$ and ${FT}^{-1}\left[\dots\right]$ denote the 2D Fourier transform and its inverse operator, respectively. The recording distance ${z}_{s}$ and the reconstruction distance ${z}_{r}$in IDH have a nonlinear relationship^32,33^. With the reconstruction distance ${z}_{r}$ set as

8 $$\begin{array}{c}{z}_{r}=\frac{\left({z}_{s}{f}_{1}+{z}_{h}{f}_{1}-{z}_{s}{z}_{h}\right)\left({z}_{s}{f}_{2}+{z}_{h}{f}_{2}-{z}_{s}{z}_{h}\right)}{\left({z}_{s}{f}_{2}+{z}_{h}{f}_{2}-{z}_{s}{z}_{h}\right)\left({f}_{1}-{z}_{s}\right)-\left({z}_{s}{f}_{1}+{z}_{h}{f}_{1}-{z}_{s}{z}_{h}\right)\left({f}_{2}-{z}_{s}\right)}\end{array}$$

a focused image of an object positioned at the recording distance ${z}_{s}$can be obtained. Although these recording and reconstruction processes are described for a single point source for simplicity, they can be straightforwardly extended to any 3D objects. An arbitrary 3D object with incoherent light is a collection of infinitesimal point sources, so its hologram and reconstructed image are created via the incoherent summation of each point source, which may be mathematically represented through 3D integration^1^.

## Design and evaluation of 1D phase grating

### Design

**Fig. 2:**
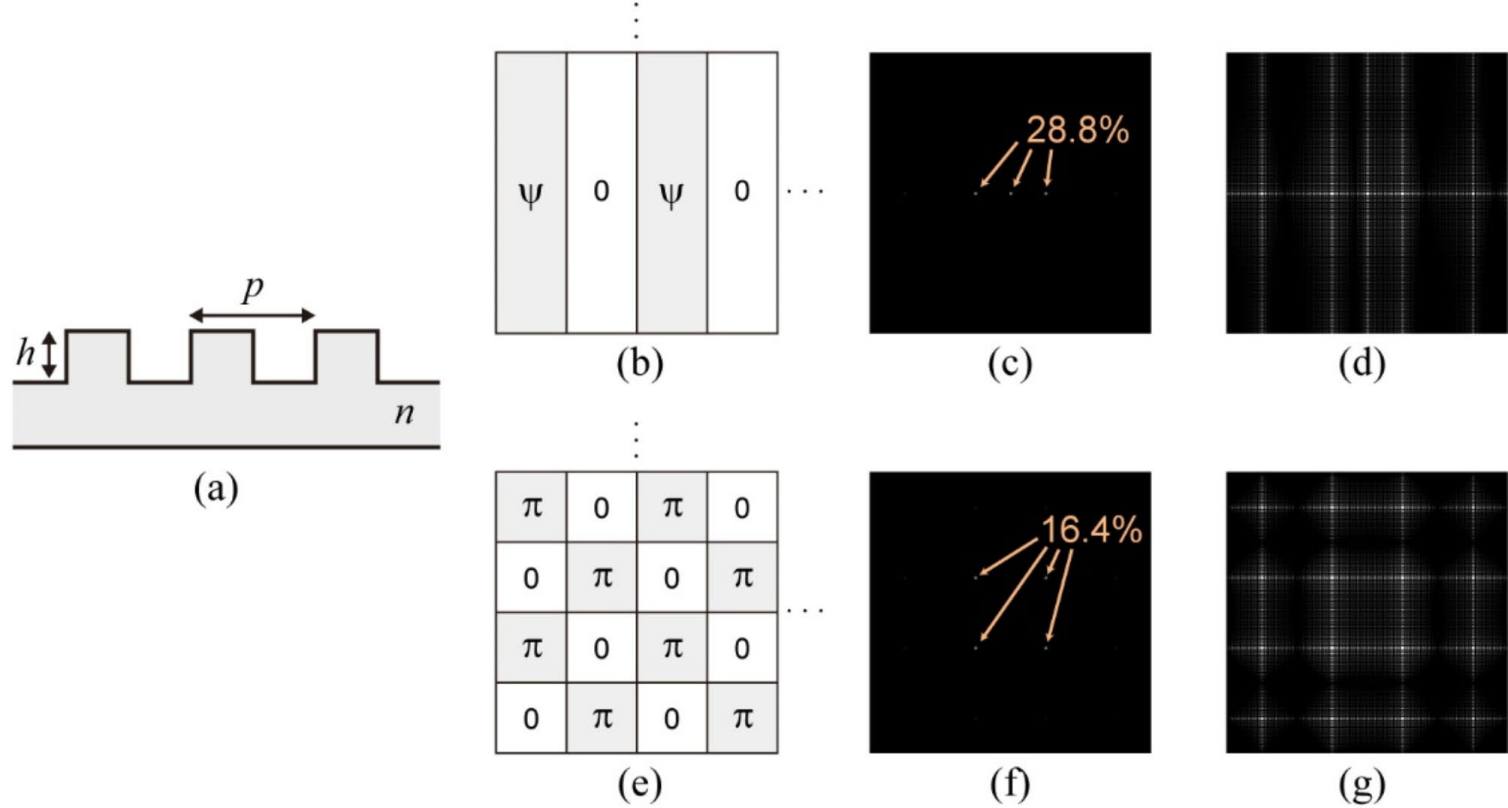
Design and comparison of phase gratings. (**a**) Design parameters for 1D phase grating. (**b**) Phase distribution, (**c**) Fourier power spectrum, and (**d**) logarithmic-scale Fourier power spectrum for designed 1D phase grating. (**e**) Phase distribution, (**f**) Fourier power spectrum, and (**g**) logarithmic-scale Fourier power spectrum for conventional 2D phase grating.

Because the 0th and ± 1st diffraction orders are used to create the three holograms, these diffraction efficiencies should be maximized. Furthermore, these diffraction efficiencies should be identical to reduce noise effectively using the phase-shifting algorithm. The 1D phase grating was set as a line-and-space structure with a unity duty cycle for the simplicity of its design and fabrication, as shown in Fig. 2(a). Under the assumption of scalar diffraction theory, the theoretical diffraction efficiencies of the 0th and ± 1st diffraction orders of a binary 1D phase grating, ${\eta}_{-1}$, ${\eta}_{0}$, and ${\eta}_{1}$, are

9 $$\begin{array}{c}{\eta}_{-1}={\text{s}\text{i}\text{n}\text{c}}^{2}\left(-\frac{1}{2}\right)\frac{{\text{s}\text{i}\text{n}\text{c}}^{2}\left(-1-\frac{\psi}{\pi}\right)}{{\text{s}\text{i}\text{n}\text{c}}^{2}\left(\frac{-\pi-\psi}{2\pi}\right)}\end{array}$$

10 $$\begin{array}{c}{\eta}_{0}=\frac{{\text{s}\text{i}\text{n}\text{c}}^{2}\left(-\frac{\psi}{\pi}\right)}{{\text{s}\text{i}\text{n}\text{c}}^{2}\left(\frac{-\psi}{2\pi}\right)}\end{array}$$

11 $$\begin{array}{c}{\eta}_{+1}={\text{s}\text{i}\text{n}\text{c}}^{2}\left(\frac{1}{2}\right)\frac{{\text{s}\text{i}\text{n}\text{c}}^{2}\left(1-\frac{\psi}{\pi}\right)}{{\text{s}\text{i}\text{n}\text{c}}^{2}\left(\frac{\pi-\psi}{2\pi}\right)}\end{array}$$

where $\psi$ is the phase difference induced by the line-and-space structure (Fig. 2(b))^29^. By sweeping the phase difference $\psi$ and numerically evaluating the above equations, we found that the phase difference $\psi$ = 0.6391π provided the same diffraction efficiency of 28.8% for the 0th and ± 1st diffraction orders. Given a wavelength $\lambda$ and the refractive index $n$ of a material, the phase difference $\psi$ could be induced on a light by properly determining the height of the line-and-space structure according to the optical path length, or $\psi=2\pi{\lambda}^{-1}\left(n-1\right)h$. For our proof-of-principle demonstration, we designed the 1D phase grating for a wavelength of 633 nm; this long wavelength allowed us to implement the light-splitting function easily due to its large diffraction angle, as described in a subsequent section. The selected material was fused silica ($n$ = 1.467 @ 633 nm), which is often used as a diffractive optical element (DOE). Under the abovementioned conditions, a phase difference $\psi$ = 0.6391π could be achieved at a height of 433 nm. Figure 2(c) shows the Fourier power spectrum of the designed 1D phase grating, where 0th and ± 1st diffraction orders are apparent, each with a diffraction efficiency of 28.8%. Because only the three main peaks contribute to creating holograms, the total light-utilization efficiency of the proposed method is 86.4% ( = 3 × 28.8%). The rest of the light energy, 13.6% ( = 100 − 86.4%), was distributed as higher diffraction orders along the horizontal direction, as shown in the logarithmic-scale Fourier power spectrum in Fig. 2(d). For comparison purposes, we included a conventional 2D phase grating^25–27^. Its Fourier power spectrum, as shown in Fig. 2(e)–2(g), lacks the 0th diffraction order and consists of four main peaks, each with a diffraction efficiency of 16.4%. The total light-utilization efficiency of the conventional method is thus 65.6% ( = 4 × 16.4%). As shown in Fig. 2(f) and 2(g), higher diffraction orders appear throughout the 2D region, resulting in a 34.4% ( = 100 − 65.6%) energy loss. Therefore, the 1D phase grating increases the light-utilization efficiency compared with the 2D phase grating, which is advantageous for recording high-quality holograms due to the high SNR. However, for simplicity, the above comparison does not consider the Fresnel loss on the grating surfaces.

Determining the grating period $p$ has two main requirements: the created holograms must have enough separation distance to prevent overlapping, and the assumption of scalar diffraction theory^29^ must hold. Given that the diffraction angle of the grating is determined by the grating period, the separation distance $s$ of the three holograms on the image sensor plane is

12 $$\begin{array}{c}s={z}_{h}\text{tan}\left[{\text{sin}}^{-1}\frac{\lambda}{p}\right].\end{array}$$

**Fig. 3:**
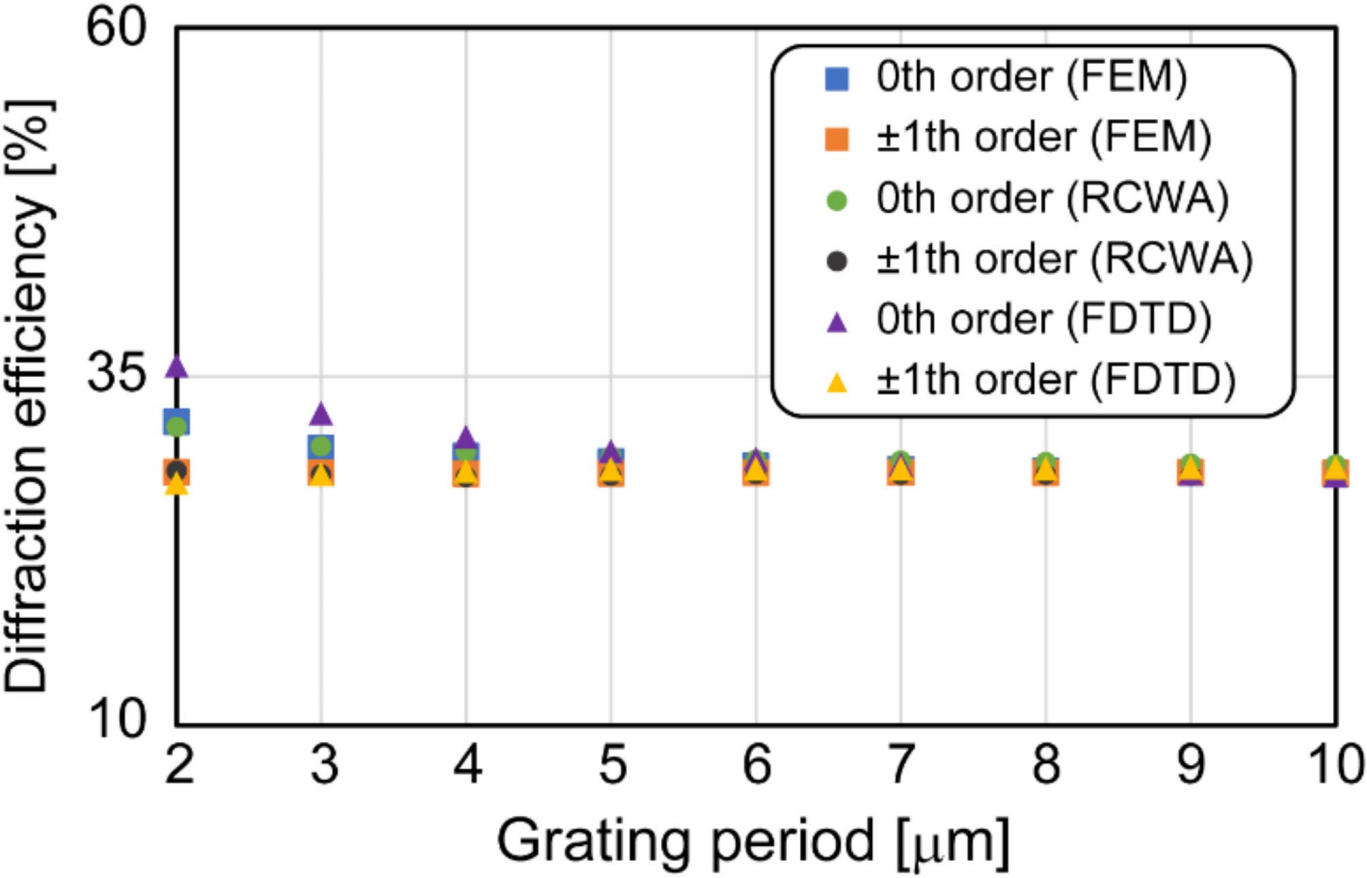
Dependence of decreasing grating period on diffraction efficiencies of 0th and ± 1st diffraction orders.

If the separation distance is smaller than the hologram diameter, the three created holograms will spatially overlap each other, so they cannot be cropped individually and processed using the phase-shifting algorithm. The separation distance should be large to prevent the holograms from overlapping. According to Eq. (12), although increasing the distance ${z}_{h}$ effectively enlarges the separation distance, it makes the optical setup bulky. The grating period $p$ should be as small as possible to develop a compact optical setup. However, an excessively small grating period $p$ does not satisfy the assumption of scalar diffraction theory. This leads to unbalanced diffraction efficiencies between the three peaks and the deformation of the created holograms. To determine the grating period, we numerically evaluated the change in diffraction efficiencies in different grating periods using numerical solvers, as shown in Fig. 3. We used three different solvers to avoid dependence on a numerical solver: the finite-difference time-domain method (Ansys Lumerical FDTD), rigorous coupled wave analysis (VirtualLab), and the finite element method (COMSOL Multiphysics). We numerically modeled the 1D phase grating with $n$=1.467 and $h$=443 nm under $\lambda$ = 633 nm illumination and regarded the grating period $p$ as a variable. Regardless of the adopted numerical solver, the numerical results show that the difference in diffraction efficiencies between the 0th and ± 1st diffraction orders increases with a decrease in the grating period. The discrepancy in the diffraction efficiencies in Fig. 3 between the numerical solvers may be due to the mesh setting and their algorithms. Knowing the best way to evaluate the diffraction efficiency is beyond the scope of this work. Although a small grating period may be acceptable for IDH, we adopted a grating period of 8 μm in our proof-of-principle experiments to satisfy the assumption of scalar diffraction theory.

### Evaluation

**Fig. 4:**
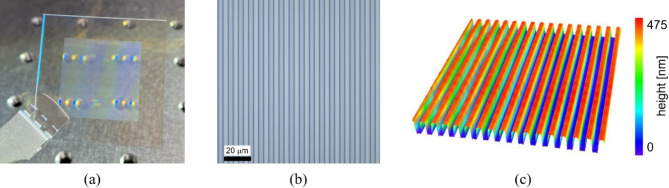
Fabricated 1D phase grating. (**a**) Photograph, (**b**) microscope image, and (**c**) 3D profile of fabricated grating.

The designed 1D phase grating was fabricated as a DOE through photolithography and then evaluated. Figure 4(a) shows a photograph of the fabricated 32 mm^2^ DOE, consisting of a line-and-space structure in a 20 mm^2^ central area. Figure 4(b) and 4(c) show an optical microscope image and a 3D profile captured using a confocal laser microscope. We evaluated the heights of five regions in the fabricated sample from the measurement result of the confocal laser microscope: middle, upper left, upper right, lower left, and lower right. The average height was 413 nm, with a fabrication error of 4.7%. Furthermore, we optically assessed the diffraction efficiencies of the fabricated sample. The sample was illuminated using a He–Ne laser with a wavelength of 633 nm. The intensities of the incident and diffracted beams were measured. By dividing the intensity of the diffracted beam by that of the incident beam, we obtained the diffraction efficiencies ${\eta}_{-1}$, ${\eta}_{0}$, and ${\eta}_{1}$. The diffraction efficiencies of the 0th- and ± 1st-diffraction-order beams are 32%, 27%, and 27%, respectively, which differ from the theoretical diffraction efficiency of 28.8%. In particular, the 0th-diffraction-order beam is more intense than the ± 1st-diffraction-order beam. This difference is mainly caused by the DOE fabrication error. The intensity of the 0th-order beam is generally difficult to control faithfully because this beam is determined by the integration of the whole DOE area^34,35^. Thus, the intensity of the 0th-order beam is sensitive to the fabrication error. This is a disadvantage of the proposed method (which has a 1D phase grating) against the conventional method^25–27^, where only the ± 1st diffraction orders from a 2D phase grating are used. The unbalanced diffraction efficiencies of the 0th- and ± 1st-diffraction-order beams of the 1D phase grating are generally undesirable for implementing the phase-shifting method. However, this problem can be mitigated by our proposed phase-shifting algorithm, expressed as Eq. (6). Moreover, despite the discrepancy between the experimental and theoretical results, the total diffraction efficiency of the proposed method (86%) is higher than that of the conventional method (65.6% in theory), which has a 2D phase grating. In the following section, we develop an IDH setup with the fabricated DOE.

## Proof-of-principle experiments

**Fig. 5:**
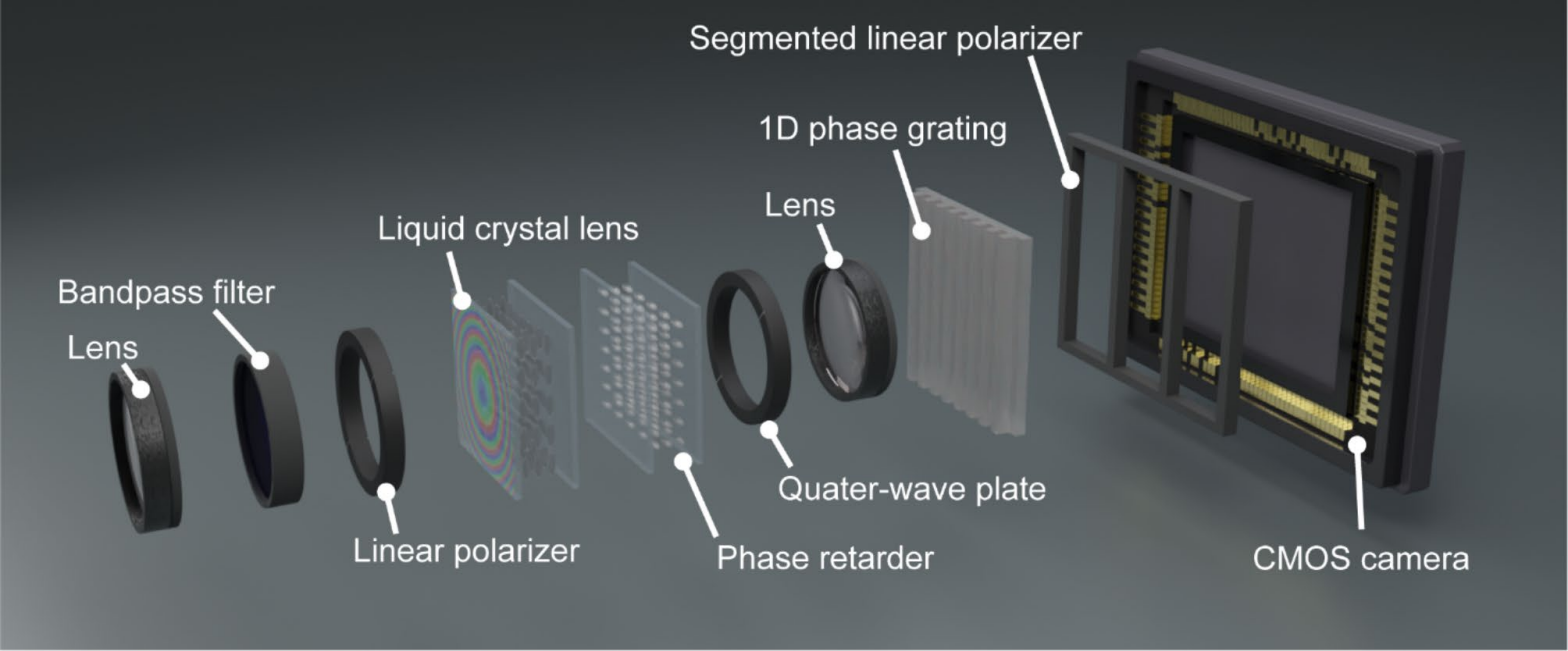
Optical setup for proof-of-principle experiment.

**Fig. 6:**
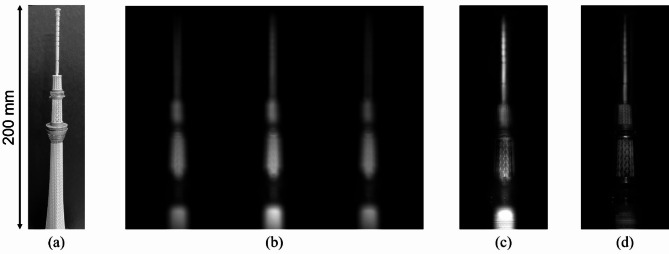
Snapshot recording results. (**a**) Captured object. (**b**) Captured single image. Reconstructed images obtained via (**c**) conventional and (**d**) proposed generalised three-step phase-shifting algorithms.

To verify the basic operation of the proposed method, we performed proof-of-principle experiments using the optical setup shown in Fig. 5. Although the concept of the optical setup was designed based on Fig. 1, optical elements were added to it. A 10 nm bandpass filter centered at 633 nm was used to enhance the temporal coherence of light, thus improving the spatial resolution of reconstructed images^36–38^. We used a liquid crystal lens^38,39^with 5.5 m and infinite focal lengths as the birefringent lens for the orthogonal linear polarizations. To achieve high spatial resolution in 3D space, large difference between two focal lengths of the birefringent lens is preferable. If we use a phase-only spatial light modulator, it is possible to implement the birefringent lens with the large difference between two focal lengths. However, to use the phase-only spatial light modulator, the optical setup should be designed on the basis of a reflective geometry with a beam splitter^27^. This leads to low light utilization efficiency. In contrast to the phase-only SLM, the ues of the liquid crystal lens allows us to develop a transmissive geometry, which increases the light utilization efficiency. Moreover, we added a liquid-crystal-based phase retarder to eliminate the optical-path difference between the interference lights^36–38^. A pair of lenses was introduced to control the image magnification^24^. For the 1D phase grating, we used the DOE described in the previous section. The segmented linear polarizer consisted of three linear polarizers whose transmission angles were rotated at $\theta=$ 0, 60, and 120 degrees. A complementary metal–oxide–semiconductor (CMOS) camera with 14,192 × 10,640 pixels and a pixel pitch of 3.76 μm was used to capture the holograms. The hologram of the object shown in Fig. 6(a) was recorded using this optical setup.

Figure 6(b) shows the image captured using the CMOS camera, which contains three individual holograms. They were cropped from the captured image to obtain the three holograms with different phase shifts. We applied the proposed phase-shifting algorithm (Eq. (6)) to the three cropped holograms. For comparison purposes, we also applied the conventional phase-shifting algorithm^30^, where the light intensity of the three holograms were assumed identical. After applying the phase-shifting algorithm, we obtained a reconstructed image via numerical back-propagation based on the angular spectrum method. Figure 6(c) and 6(d) show the reconstructed images obtained by the conventional and proposed phase-shifting algorithms, respectively. The reconstructed image in Fig. 6(c) has dim background noise resulting from the intensity variations between the three holograms due to the fabrication error of the 1D phase grating, as discussed in the previous section. A part of the background noise interferes with the object light, leading to the reverse contrast on the reconstructed image. The reconstructed image in Fig. 6(d) has less background noise because the intensity variations between the three holograms are compensated by the proposed phase-shifting algorithm. The snapshot results show that the proposed method enables the single-exposure recording of the holograms. Moreover, our phase-shifting algorithm removes the intensity variations caused by the fabrication error of the 1D phase grating.

**Fig. 7:**
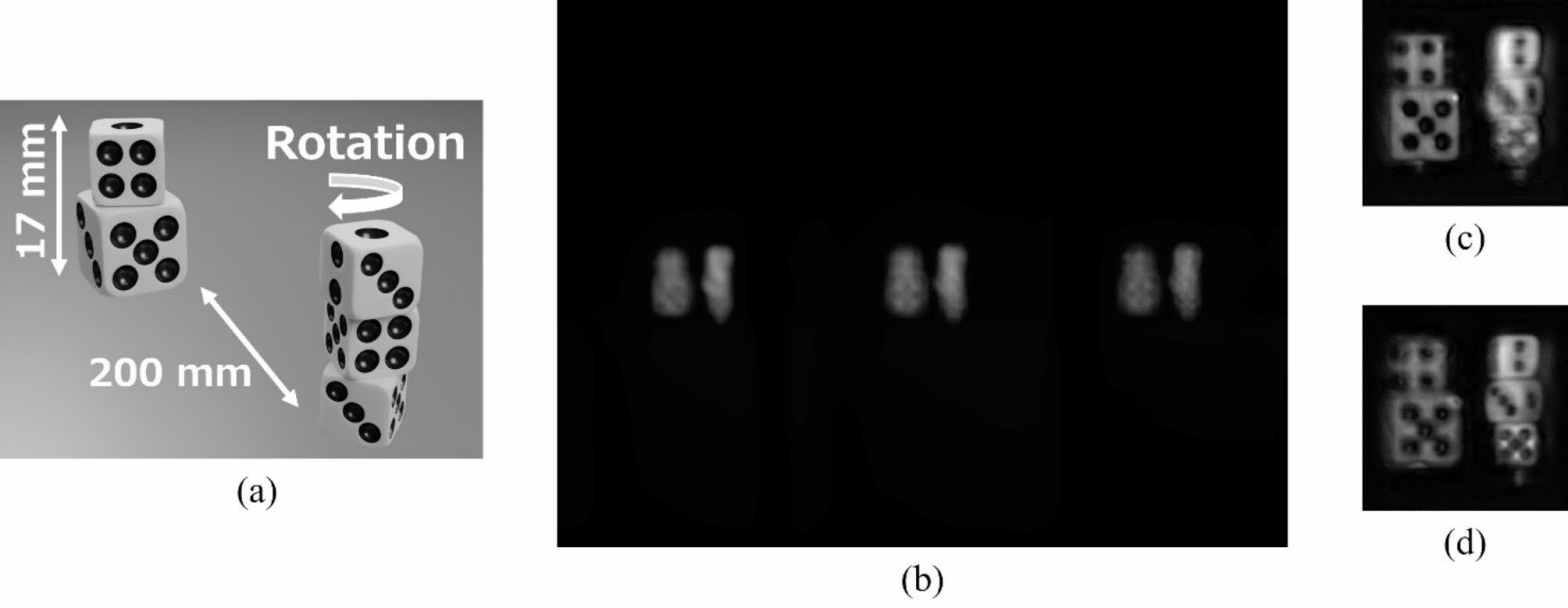
Video recording results. (**a**) Illustration of captured objects. (**b**) Captured single image. Reconstructed images focused on (**c**) back and (d) front objects.

To further verify the proof-of-concept of the proposed method, we experimentally demonstrated a video recording of the holograms. The captured objects were two stacks of dice that were axially separated by 200 mm (Fig. 7(a)). The front stack was placed on a rotating mechanical stage. The LED lighting and optical setup were the same as those used in the previous experiment (snapshot recording). We recorded 50 frames at approximately 2 fps. Figure 7(b) shows one frame of the recorded holograms. By processing the recorded holograms through cropping, proposed generalised three-step phase-shifting, and numerical propagation, we obtained the reconstructed images in Fig. 7(c) and 7(d), which focus on the front and back objects, respectively. Note that there is the difference of the image quality between Fig. 7(c, d) and Fig. 6(d) because the hologram contrast of IDH is affected by the size and sparsity of objects to be captured. Supplementary Video 1 is a video of the reconstructed images from all recorded frames. The rotating dice were successfully captured with the proposed method. For comparison purposes, we obtained a video of the reconstructed images with the conventional three-step phase-shifting method as shown in Supplementary Video 2. In this video, it is difficult to verify the object image. These results show that the proposed method has 3D imaging and refocusing capabilities in each frame, enabling the recording of 3D videos.

## Discussion

According to the results, the 1D phase grating can achieve higher light-utilization efficiency compared with the 2D phase grating. High light-utilization efficiency is preferable for recording high-quality holograms with high SNRs during hologram detection with an image sensor. However, phase shifting with the 1D phase grating retrieves the complex amplitude from the three holograms; the noise tolerance during this retrieval process may be lower than that in the conventional four-step phase-shifting method with a 2D phase grating, because the more phase-shifted holograms are used, the better the robustness^40^. This illustrates a trade-off between the light-utilization efficiency and noise tolerance in grating-assisted space-division phase shifting. To reveal the merit of the proposed method with the 1D phase grating, we theoretically compared the performance of four- and three-step phase-shifting methods for grating-assisted space-division phase shifting.

The four-step phase-shifting method retrieves the complex amplitude ${u}_{\text{F}}$ through

13 $$\begin{array}{c}{u}_{\text{F}}=\frac{1}{4}\left\{\text{exp}\left(i\cdot0\right){I}_{\text{F}0}+\text{exp}\left(i\cdot\frac{\pi}{2}\right){I}_{\text{F}1}+\text{exp}\left(i\cdot\pi\right){I}_{\text{F}2}+\text{exp}\left(i\cdot\frac{3\pi}{2}\right){I}_{\text{F}3}\right\}.\end{array}$$

In this equation, each hologram with the shot noise captured using an image sensor is denoted as

14 $$\begin{array}{c}{I}_{\text{F}m}=B+\text{e}\text{x}\text{p}\left(-i{\theta}_{m}\right)o+\text{e}\text{x}\text{p}\left(i{\theta}_{m}\right){o}^{*}+{n}_{\text{F}m},\end{array}$$

where $B$ is the bias term of the holograms and ${\theta}_{m}$ is the phase-shift amount. We simply model the shot noise ${n}_{\text{F}m}$ as

15 $$\begin{array}{c}{n}_{\text{F}m}\sim N\left(0,{\frac{1}{{\eta}_{\text{F}}}\sigma}^{2}\right),\end{array}$$

where $N\left(0,\sigma\right)$ is a zero-mean Gaussian distribution with a standard deviation $\sigma$. ${\eta}_{\text{F}}$ denotes the diffraction efficiency of each diffraction order of the 2D phase grating. The phase grating and optical setup are assumed to be ideally fabricated and constructed, eliminating variations between the diffraction efficiencies and undesired phase shifts. By substituting Eq. (14) into Eq. (13), we rewrite Eq. (13) as

16 $$\begin{array}{c}{u}_{\text{F}}=o+\frac{1}{4}\left[{n}_{\text{F}0}-{n}_{\text{F}2}+i\left({n}_{\text{F}1}-{n}_{\text{F}3}\right)\right]=o+{n}_{\text{F}}.\end{array}$$

According to the reproductive property of the normal distribution, the probability density function of ${n}_{\text{F}}$ is

17 $$\begin{array}{c}{n}_{\text{F}}\sim\:CN\left(0,{\frac{1}{4{\eta}_{\text{F}}}\sigma}^{2}\right),\end{array}$$

where $\mathcal{C}\mathcal{N}(.,.)$denotes a circular complex Gaussian distribution^41^. This shows that the four-step phase-shifting method suffers from complex Gaussian noise with a variance ${\left(4{\eta}_{\text{F}}\right)}^{-1}{\sigma}^{2}$. An increase in the diffraction efficiency ${n}_{\text{F}}$ reduces the noise variation. By contrast, for the three-step phase-shifting method, the complex amplitude is obtained from the three holograms.

18 $$u_T=\frac{1}{4}\{\text{exp}(i\cdot0){I}_{\text{T}0}+\text{exp}(i\cdot\frac{2\pi}{3}){I}_{\text{T}1}+\text{exp}(i\cdot\frac{4\pi}{3}){I}_{\text{T}2}\}$$

19 $$\begin{array}{c}{I}_{\text{T}m}=B+\text{e}\text{x}\text{p}\left(-i{\theta}_{m}\right)o+\text{e}\text{x}\text{p}\left(i{\theta}_{m}\right){o}^{*}+{n}_{\text{T}m}.\end{array}$$

We also simply model the shot noise ${n}_{\text{T}m}$ with the Gaussian distribution, or

20 $$\begin{array}{c}{n}_{\text{T}m}\sim N \left(0,{\frac{1}{{\eta}_{\text{T}}}\sigma}^{2}\right),\end{array}$$

where ${\eta}_{\text{T}}$ denotes the diffraction efficiency of each diffraction order of the 1D phase grating. By substituting Eq. (19) into Eq. (18), we rewrite Eq. (18) as

21 $$\begin{array}{c}{u}_{\text{T}}=o+\frac{1}{3}\left[{n}_{\text{F}0}-\frac{1}{2}{n}_{\text{F}1}-\frac{1}{2}{n}_{\text{F}2}+i\left({\frac{\sqrt{3}}{2}n}_{\text{F}1}-\frac{\sqrt{3}}{2}{n}_{\text{F}2}\right)\right]=o+{n}_{\text{T}}.\end{array}$$

According to the reproductive property of the normal distribution, the probability density function of ${n}_{\text{T}}$ is

22 $$\begin{array}{c}{n}_{\text{T}}\sim\:CN\left(0,{\frac{1}{3{\eta}_{\text{T}}}\sigma}^{2}\right).\end{array}$$

This shows that the three-step phase-shifting method suffers from complex Gaussian noise with a variance ${\left(3{\eta}_{\text{T}}\right)}^{-1}{\sigma}^{2}$. To compare the four- and three-step phase-shifting methods, we introduce the noise ratio as a metric.

23 $$\begin{array}{c}{NR}_{T/F}=\frac{{\left(3{\eta}_{\text{T}}\right)}^{-1}{\sigma}^{2}}{{\left(4{\eta}_{\text{F}}\right)}^{-1}{\sigma}^{2}}=\frac{4{\eta}_{\text{F}}}{3{\eta}_{\text{T}}}\end{array}$$

This equation contains the effects of the differences in light-utilization efficiency of the phase gratings and the noise tolerances determined by the number of phase-shifted holograms during the implementation of the phase-shifting algorithm. When ${NR}_{T/F}$ = 1, the reconstructed images under the four- and three-step phase-shifting methods have the same amounts of noise. When ${NR}_{T/F}$ < 1, the three-step phase-shifting method is preferable and can reduce noise better than the four-step phase-shifting method, and vice versa. Thus, ${\eta}_{\text{F}}/{\eta}_{\text{T}}$ should be lower than three quarters. Given the theoretical diffraction efficiencies ${\eta}_{\text{F}}$ and ${\eta}_{\text{T}}$ of 16.4% and 28.8%, respectively, ${\eta}_{\text{F}}/{\eta}_{\text{T}}\cong\:0.57$, showing the advantage of the proposed method in terms of the noise amount. However, the diffraction efficiencies in this work are for binary phase gratings. In the case of multilevel phase gratings, the diffraction efficiencies of 2D and 1D phase gratings can reach 25% and 33.3…%, respectively. Consequently, ${NR}_{T/F}=1$, which means that the gain from the higher light-utilization efficiency and the noise reduction due to the higher-step phase-shifting method are totally conserved. Therefore, if it is possible to fabricate and use the multilevel phase grating, the performance of the grating-assisted spatial phase-shifting method is not affected by the number of divided beams. However, the fabrication of multilevel gratings with acceptable fabrication errors is practically challenging. In addition to DOEs, metasurfaces^42,43^ may be an effective approach to fabricating multilevel phase gratings because metasurfaces can implement multilevel phase modulation with one-step structures and changes in their in-plane dimensions.

## Conclusion

We proposed and developed a single-shot IDH system based on generalised three-step phase shifting with a 1D phase grating. The 1D phase grating can achieve higher light-utilization efficiency than the 2D phase grating used in the conventional method. This can increase the frame rate of video recording or the quality of reconstructed images. The disadvantage of the 1D phase grating is the variation in the diffraction efficiencies of the 0th and ± 1st diffraction orders, as controlling the 0th-order intensity is technically difficult due to DOE fabrication errors. This difficulty of DOE fabrication can be compensated by our generalised three-step phase-shifting algorithm. Our proposed method shows a novel direction (3D videography) for implementing single-shot phase-shifting IDH systems.

We also theoretically compared the performance of the three-step phase-shifting method with the 1D phase grating and the four-step phase-shifting method with the 2D phase grating for grating-assisted space-division phase shifting. The theoretical investigations show that when the gratings are designed based on binary modulation, the 1D phase grating is preferable in terms of noise tolerance. As for the design and fabrication of 1D and 2D phase gratings with multilevel modulation, the four- and three-step phase-shifting methods have the same noise tolerances. This theoretical finding is acceptable, as the energy of the input light on the grating is the same, regardless of the number of divided beams with the grating. However, the fabrication of multilevel gratings is challenging. Fabricating 1D or 2D phase gratings with multilevel phases can further improve the imaging performance of grating-assisted space-division phase shifting.

## Electronic supplementary material

Below is the link to the electronic supplementary material.

Supplementary Material 1

Supplementary Material 2

## Data Availability

The data that support the findings of this study are available from the corresponding author upon reasonable request.
